# Medium-term outcomes of 78,808 patients after heart valve surgery in a middle-income country: a nationwide population-based study

**DOI:** 10.1186/s12872-017-0725-9

**Published:** 2017-12-28

**Authors:** Regina Maria de Aquino Xavier, Vitor Manuel Pereira Azevedo, Paulo Henrique Godoy, Arn Migowski, Antonio Luiz Pinho Ribeiro, Rogério Brant Martins Chaves, Marcelo Goulart Correia, Carolina de Aquino Xavier, Lucas de Aquino Hashimoto, Clara Weksler, Nelson Albuquerque Souza e Silva

**Affiliations:** 1 Hospital School, National Institute of Cardiology (INC), Ministry of Health, Rua das Laranjeiras 374, Laranjeiras, Rio de Janeiro, RJ Brazil; 2 Hospital Naval Marcílio Dias, Rio de Janeiro, Brazil; 3 University Hospital and School of Medicine - Universidade Federal de Minas Gerais (UFMG), Minas Gerais, Brazil; 4 University Hospital and School of Medicine, Federal University of Rio de Janeiro (UFRJ), Rio de Janeiro, Brazil; 5 Hospital Municipal Salgado Filho, Rio de Janeiro, Brazil

**Keywords:** Cardiac surgical procedures, Heart valve diseases, Survival analysis, Hospital mortality, Rheumatic heart disease, Medical record linkage, Brazil, Annular repair, cardiac valve

## Abstract

**Background:**

Heart valve surgery outcomes are unknown in middle-income countries and thus cannot be used in health system decision making processes. This study estimated in-hospital mortality and medium and long-term survival.

**Methods:**

This was a retrospective study of 78,806 patients who underwent heart valve surgery between 2001 and 2007 in Brazil. Two national databases were used, the Hospital Information System and the Mortality Information System. Kaplan-Meier survival analysis and log-rank tests were performed. Maximum and median follow-up was 7.7 and 2.8 years, respectively (0.002–7.707).

**Results:**

Valve replacement accounted for 69.1% of procedures performed. Mitral stenosis, the most common valve injury, represented 38.9% of the total. In 94.7% of mitral stenosis patients, aetiology was rheumatic heart disease. In-hospital mortality was 7.6% and was higher for women, for patients who had undergone concomitant coronary artery bypass grafting (CABG) and for the elderly. Overall survival was 69.9% at the end of follow-up. Survival was worst among elderly, male and concomitant CABG patients (*P*<0.001).

**Conclusions:**

Rheumatic heart disease is still a major public health problem in Brazil. In-hospital mortality and global survival rates of patients who have undergone heart valve surgery were less satisfactory than those reported in high-income countries. The findings of this study can contribute to guiding decision making processes in middle-income countries similar to Brazil and others concerned with improving the quality of care.

## Background

In low and middle-income countries, heart valve disease (HVD) is caused mainly by rheumatic heart disease (RHD) [1–6]. Although incidence of acute rheumatic fever has been declining over recent decades in some of these countries, RHD is still considered a major public health problem worldwide [5]. In high-income countries, HVD remains common, despite decreasing prevalence of RHD, because the decline in inflammatory HVD aetiology has been accompanied by an increase in degenerative valve diseases [7]. Most studies describing HVD patterns have been performed in developed countries, which contain less than 20% of the world’s population [4]. Large-scale nationwide studies in developing countries are scarce [4, 5].

In Brazil, although the incidence of acute rheumatic fever has been reduced in recent decades [8], a large number of patients need to perform heart valve surgery (HVS) to repair or replace valves that have suffered rheumatic injury. In these cases, the mitral or aortic valves, or both simultaneously, are the most affected [9]. Moreover, as in high-income countries, population ageing has led to an increased number of injuries caused by cardiac valve degeneration. As a result, Brazil has a double burden of diseases connected with poverty, such as rheumatic fever, and degenerative diseases associated with an aging population [10].

Little is known about HVS patient survival in low- and middle-income countries. Most surgical risk evaluations have been based on high-income country data. Similarly, health managers who need to allocate scarce resources rationally to achieve quality in health services are severely challenged. Moreover, disclosure of variations in cardiac surgery outcomes is known to offer opportunities to improve the quality of health care and to be an essential tool for quality assessment.

This study aims to estimate in-hospital mortality, long-term survival and average length of hospital stay of patients submitted to HVS between 2001 and 2007, from data in the nationwide database of the Brazilian National Health System (*Sistema Único de Saúde, SUS*).

## Methods

### Study setting and population

This was a retrospective nationwide cohort study of 78,808 consecutive patients who underwent HVS in the SUS from 2001 to 2007. The SUS has 234 hospitals with cardiovascular surgery. It provides universal care for over 200 million people [11] and is the sole coverage for 70% of Brazil’s population [12].

In the SUS, hospital records have been processed to an administrative database, the Hospital Information System (*Sistema de Informação Hospitalar*, SIH), since 1979, using International Classification of Diseases (ICD) codes. Mortality information since 1975 is on record in a Mortality Information System (*Sistema de Informação de Mortalidade*, SIM) that covers the whole population nationwide [13]. This mortality data is considered qualitatively reliable and as accurate as that of other countries with long traditions in these statistics [14]. The accuracy of the SIH variables for diagnosis, medical procedures, sex, age group and in-hospital outcomes is considered satisfactory [15].

The study cohort comprised patients submitted to one of the following *SUS* surgical procedures, as they are labelled in the SIH database, from 2001 to 2007:

- Valve replacement (VRp): replacing a heart valve with a biological or mechanical prosthesis;
- Valve repair (VRr): restoring the normal functioning of heart valves without the use of a prosthesis; may use a support ring;
- Multiple valve repair and/or replacement (multiple valve surgeries): repair and/or replacement of more than one heart valve (mitral, aortic, tricuspid or pulmonary) with biological or mechanical prostheses;
- Valve replacement with concomitant coronary artery bypass grafting (concomitant CABG): mitral or aortic valve replacement by biological or mechanical prosthesis and concomitant CABG.

All the HVSs described above were performed by thoracotomy and cardiopulmonary bypass.

We did not select any procedure related exclusively to congenital heart diseases.

The exclusion criteria were the following: records with name field left blank or filled beginning with the words “woman”, “man”, “stillbirth”, “son”, “daughter”, “newborn” or “unknown” and records with date of birth left blank. After cleaning the SIH and SIM databases, 5026 (6.0%) records were excluded.

Patients in the concomitant CABG group displayed different demographic characteristics from other patients: they were older, entailing degenerative processes towards valvular lesion (although in developing countries, such as Brazil, rheumatic disease cannot be precluded as a concomitant process). Moreover, it should not be forgotten that valve surgery and CABG are two separate procedures, thus extracorporeal time is extended. This group was therefore subjected to statistical sub-analysis as a separate cohort.

The inpatient database provides the following information for each individual patient receiving care: ICD 10.0 code, date of birth, date of admission, date of discharge and date of death (if appropriate), sex, type of surgical procedure, disease by valve, length of stay, discharge status, geographical region of residence and county of residence at time of admission. This database was initially set up for administrative purposes, although several studies have validated its use for research purposes [16]. The death certificate provides basic cause of death (ICD 10.0 code), plus demographic data, such as name, dates of birth and death, geographical region of residence and county of residence and of death.

### Study measures

The outcome measures for this study were in-hospital mortality (defined as death during index admission) and time until death during 7-year follow-up. These measures were obtained by probabilistic record linkage [17] using the SIH and SIM databases. The linkage method applied showed 90.6% sensitivity and 100% specificity [18] and was used in a prior study [19].

Patient follow-up was considered for discharges from 1 January 2001, while the cut-off entry date for new records was 30 November, 2007, in order to ensure at least a possible 30 days of observation for all patients in this study. Patients were followed up until death or were censored at 31 December 2007, the last day of the study period.

Probabilistic linkage was performed using the public domain software, RecLink III version 3.1 [17].

### Statistical analysis

Descriptive analysis included calculating mean, standard deviation, median and interquartile range (IQR) for continuous variables. Categorical variables were described by counts and relative frequency. Continuous variables were compared between dichotomous groups using Student’s t-test for normal distributions and Mann-Whitney, otherwise. For continuous variables with more than two groups and non-normal distribution, the Kruskal-Wallis test was employed. The chi-square test was used to compare categorical variables. The Kolmogorov-Smirnov test rejected the null hypothesis for normal age distribution, therefore the median and interquartile range were used (*p* < 0.0001).

To study in-hospital mortality, univariate and multivariate logistic regression models were performed and odds ratios and their respective 95% confidence intervals (95%CI) were calculated. Multivariate testing was performed in forward stepwise fashion.

Survival curves were constructed with Kaplan-Meier techniques and compared with the log-rank statistic. The Cox proportional hazards model was used and hazard ratios (HRs) and their 95%CIs were reported. Schoenfeld residuals were used to test the proportional hazards assumption [20]. Bivariate Cox proportional hazards regression was performed, followed by multivariate models to estimate the independent effect of the variables previously selected. In the event of violation of the proportional hazard assumption, the Stratified Cox model was employed.

Analyses were performed with STATA software, version 13 (STATA Corp, College Station, Tex.). All statistical tests were two-tailed if not stated otherwise. The alpha level was set at 0.05.

## Results

Maximum and median follow-up were 7.7 and 2.8 years, respectively (0.002–7.707). The 78,808 patients included in the study were similarly distributed by sex, with 40,136 (51.0%) females and 38,702 (49.0%) males. Median age was 50.0 (35.9–62.5) years. Most were residents of Brazil’s southeast region. Median hospital stay was 10 (7–13, 16–18) days. The most common surgery procedure was VRp, in 54,475 (69.1%) patients. The baseline characteristics of the final cohort are described in Table 1.

**Table 1:** Demographic characteristics, procedures and length of stay of heart valve surgery patients – 2001 to 2007, Brazil

Variable	Patients (*n* = 78,808)
Age (years), median (IQR)	50.0 (35.9–62.5)
Age (years), % by age group	
< 20.0	6.37
20–39.9	25.25
40–59.9	38.61
60–79.9	28.63
≥ 80	1.14
Female, n (%)	40,106 (50.89)
Admission by geographical region of residence, n (%)	
Southeast	36,337 (46.11)
Northeast	14,442 (18.33)
North	2360 (2.99)
Midwest	6919 (8.78)
South	18,750 (23.79)
Length of hospital stay (days), median (IQR)	10 (7–16)
Valve repair	4989 (6.33)
Valve replacement	54,475 (69.12)
Multiple valve repair and/or replacement	12,197 (15.48)
Concomitant CABG	7147 (9.07)

*IQR* Interquartile range, Concomitant CABG – Valve replacement with concomitant coronary artery bypass graft surgery

Valve injury aetiology, when identified, is shown in Table 2. The most prevalent cause was rheumatic heart disease. The second group was classified as “insufficient aetiology description” (IAD). This group included situations when there is no specific ICD10.0 code to describe the aetiology; for example, degenerative aortic stenosis, probably coded just as “aortic stenosis”. IAD group median age was 60.7 years. Median age was lower (44.2 years) in RHD patients than in the remaining cohort (57.5 years) (*p* < 0.001). In mitral stenosis patients, aetiology was RHD in 94.7% (p < 0.001). As aetiology was improperly encoded in 35.1% of patients undergoing HVS, this variable was not used in risk assessment.

**Table 2:** Frequency and proportion of valve lesion aetiologies in heart valve surgery patients – 2001 to 2007, Brazil

Aetiology	n (%)
Rheumatic heart disease	42,352 (53.74)
Insufficient description of the aetiology	27,585 (35.00)
Infective Endocarditis	7177 (9.11)
Congenital	1354 (1.72)
Prolapsed mitral valve	187 (0.24)
Kawasaki disease or Takayasu arteritis	91 (0.12)
Endomyocardial fibrosis	38 (0.05)
Hypertrophic cardiomyopathy	24 (0.03)
Total	78,808 (100%)

In-hospital mortality was 7.6% (3585 patients) and overall mortality at close of study period was 22.1% (17,383 patients) from all causes of death. Most causes were classified by ICD codes as diseases of the circulatory system (13,449 deaths; 77.6%), followed by diseases of the respiratory system (813 deaths; 4.7%).

Table 3 shows a subset of patients (*n* = 47,515) where the type of mitral or aortic valve disease was identified and where there was no concomitant CABG. The most common valve injury encountered was mitral stenosis (38.9%). Patients with combined aortic and mitral lesions were the youngest (median 43.3 years) and those with aortic stenosis were the oldest (median 58.0 years) (*p* < 0.001). Repair was performed predominantly in females (65.9%; *p* < 0.0001).

**Table 3:** Frequency and proportion of valve disease types in non-concomitant coronary artery bypass graft surgery in heart valve surgery patients – 2001 to 2007, Brazil

Type of valve disease	n (%)
Double aortic lesion	1980 (4.17)
Double mitral lesion	2097 (4.41)
Aortic stenosis	7019 (4.77)
Mitral stenosis	18,471 (38.88)
Aortic regurgitation	5241 (11.03)
Mitral regurgitation	8985 (18.91)
Aortic + Mitral lesion	3722 (7.83)
Total	47,515

When stratified by age group, highest in-hospital mortality was observed in patients aged ≥80 years, and the lowest was for those between 20 and 39.9 years (*p* < 0.001). In-hospital mortality rates differed significantly by sex; they were higher among women (7.8% versus 7.3%; *p* < 0.001), although this difference was small (Table 4). In-hospital mortality by valve procedure was 3.5% for VRr, 6.9% for VRp, 8.2% for multiple valve repair and/or replacement and 14.6% for concomitant CABG (*p* < 0.001).

**Table 4:** Frequency and proportion of in-hospital mortality, by age group, in heart valve surgery patients – 2001 to 2007, Brazil

Age, by age group	Living, n (%)	Deceased, n (%)	Total, n
<19.9	4756 (94.78)	262 (5.22)	5018
20–39.9	19,175 (96.37)	723 (3.63)	19,898
40–59.9	28,400 (93.34)	2027 (6.66)	30,427
60–79.9	19,776 (87.64)	2788 (12.36)	22,564
≥80	735 (81.58)	166 (18.42)	901
Total	72,842 (92.43)	5966 (7.57)	78,808

Univariate and multivariate logistic regressions (Tables 5 and 6) were performed to identify independent per-operative predictors for in-hospital mortality. All variables that were significant (<0.10) in univariate analyses were added to the multivariate logistic regression model. Corresponding odds ratios (ORs) and 95%CIs were calculated. Because of their low frequency, the following aetiologies have been grouped as “other”: Kawasaki disease, Takayasu arteritis, endomyocardial fibrosis and hypertrophic cardiomyopathy.

**Table 5:** Univariate analysis by logistic regression of in-hospital mortality in heart valve surgery patients – 2001 to 2007, Brazil

Variable	OR	95% CI	*P*
Age (years)	1.0338	1.0316–1.0361	<0.001
Female	1.1410	1.0669–1.2204	<0.001
Procedures:			
Repair (reference)			
Replacement	2.2512	1.8015–2.8131	<0.001
Multiple valve repair and/or replacement	4.0268	3.1990–5.0688	<0.001
Concomitant CABG	7.1102	5.6444–8.9565	<0.001
Any valve surgery (reference)			
Concomitant CABG	2.8941	2.6606–3.1481	<0.001
Type of valve disease:			
Aortic regurgitation (reference)			
Mitral stenosis	1.1293	0.9488–1.3441	0.171
Double mitral lesion	1.1743	0.8905–1.5485	0.255
Mitral regurgitation	1.2343	1.0217–1.4912	0.029
Aortic stenosis	1.2492	1.0266–1.5200	0.026
Aortic + Mitral lesion	1.3667	1.0924–1.7097	0.006
Double aortic lesion	1.4375	1.1060–1.8684	0.007
Aetiology			
Rheumatic heart disease (reference)			
Congenital	1.4653	1.1358–1.8905	0.003
Infective Endocarditis	1.5525	1.3796–1.7470	<0.001
Other	1.8162	1.1524–2.8622	0.010
Insufficient description of aetiology	1.9407	1.8058–2.0857	<0.001

*OR* Odds ratio Concomitant CABG – Valve replacement with concomitant coronary artery bypass graft surgery

**Table 6:** Multivariate analysis, by logistic regression, for overall in-hospital mortality, by age and sex, in heart valve surgery patients – 2001 to 2007, Brazil

Procedures:	OR	95% CI	*P*
Valve repair (reference)			
Valve replacement	2.0728	1.7740–2.4219	<0.001
Multiple valve repair and/or replacement	2.5011	2.1202–2.9507	<0.001
Concomitant CABG	4,7693	4.0410–5.6288	<0.001
Any valve surgery (reference)			
Concomitant CABG	2.3070	2.1472–2.4789	<0.001
Type of valve disease:			
Aortic regurgitation (reference)			
Mitral stenosis	1.1350	0.9997–1.2886	0.051
Double mitral lesion	1.0072	0.8147–1.2451	0.947
Mitral regurgitation	1.1392	0.9907–1.3100	0.067
Aortic stenosis	1.1013	0.9513–1.2751	0.196
Aortic + Mitral lesion	1.1950	1.0089–1.4152	0.039
Double aortic lesion	1.1744	0.9577–1.4403	0.123
Aetiology			
Rheumatic heart disease (reference)			
Congenital	1.5601	1.2879–1.8899	<0.001
Infective Endocarditis	2.4386	2.2524–2.6404	<0.001
Other	3.2313	2.4241–4.3073	<0.001
Insufficient description of aetiology	1.4989	1.4137–1.5892	<0.001

*OR* odds ratio Concomitant CABG – Valve replacement with concomitant coronary artery bypass surgery

The following tables show multivariate analysis by stepwise logistic regression for in-hospital mortality including the variables age and female sex. Table 7 compares concomitant CABG with the other surgeries studied in our cohort. In this comparison, concomitant CABG increases risk (OR = 2.0366). Table 8 compares valve repair against replacement or multiple valve surgeries. In this comparison, repair reduces risk (OR = 0.4586).

**Table 7:** \Multivariate analysis, by stepwise logistic regression, for in-hospital mortality of heart valve surgery patients – 2001 to 2007, Brazil

Variables	OR	95% CI	*P*
Age (year)	1.0302	1.0279–1.0325	<0.001
Female	1.3051	1.2188–1.3975	<0.001
Concomitant CABG	2.0366	1.8643–2.2249	<0.001

*OR* Odds ratio Concomitant CABG – Valve replacement with concomitant coronary artery bypass surgery

**Table 8:** Multivariate analysis, by stepwise logistic regression, for in-hospital mortality of heart valve surgery patients, except CABG – 2001 to 2007, Brazil

Variables	OR	95% CI	*P*
Age (year)	1.0289	1.0264–1.0314	<0.001
Female	1.2776	1.1835–1.3789	<0.001
Valve repair	0.4586	0.3670–0.5731	<0.001

OR – odds ratio

Shortest hospital stays were for repair procedures (median = 9 days) (*p* < 0.001) and showed no difference when analysed by sex (*p* = 0.21). They were shorter for patients who died during index procedure admission (7 days) than for survivors (10 days) (*p* < 0.001). Significantly longer hospital stays were observed among patients who underwent concomitant CABG (11 days) and those over 80 years old (12 days).

**Fig. 1:**
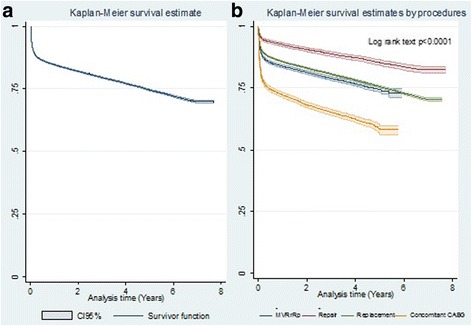
**a** Overall survival of heart valve surgery patients – 2001 to 2007; (**b**) Long-term survival of heart valve surgery patients, by procedure type

Overall survival of HVS patients is shown in Table 9 and Fig. 1a. There were 78,808 HVS patients, and survival was 69.9% (69.38–70.47%) at the end of follow-up time. Table 10 shows overall survival of patients with rheumatic heart disease. Figure 1b shows survival by procedure: at 1 and 5 years, this was 92.5% (95%CI 91.8–93) and 85.3% (95%CI 84.2–86.4) for valve repair; 85.7% (95%CI 85.4–86.0) and 75.5% (95%CI 75.1–76) for valve replacement; 83.8% (95%CI 83.1–84.4) and 74.4% (95%CI 73.4–75.5) for multiple valves surgery; and 72.2 (95%CI 71.1–73.2) and 58.1% (95%CI 56.2–60) for concomitant CABG. Survival, by type of valvular lesion showed no differences.

**Table 9:** Overall survival of heart valve surgery patients – 2001 to 2007, Brazil

Time (years)	Survival Function (%)	Standard Error (%)	95% CI (%)
1	84.62	0.13	84.37–84.87
2	81.98	0.14	81.70–82.25
3	79.49	0.15	79.19–79.78
4	77.12	0.17	76.79–77.44
5	74.54	0.18	74.18–74.90
6	72.06	0.21	71.64–72.47
7	69.93	0.28	69.38–70.47

**Table 10:** Overall survival of heart valve surgery patients with rheumatic heart disease – 2001 to 2007, Brazil

Time (years)	Survival Function (%)	Standard Error (%)	95% CI (%)
1	93.73	0.12	93.48–93.97
2	91.55	0.14	91.27–91.83
3	89.57	0.17	89.25–89.89
4	87.50	0.19	87.13–87.87
5	85.24	0.22	84.80–85.67
6	83.07	0.28	82.52–83.61
7	81.35	0.40	80.55–82.11

**Fig. 2:**
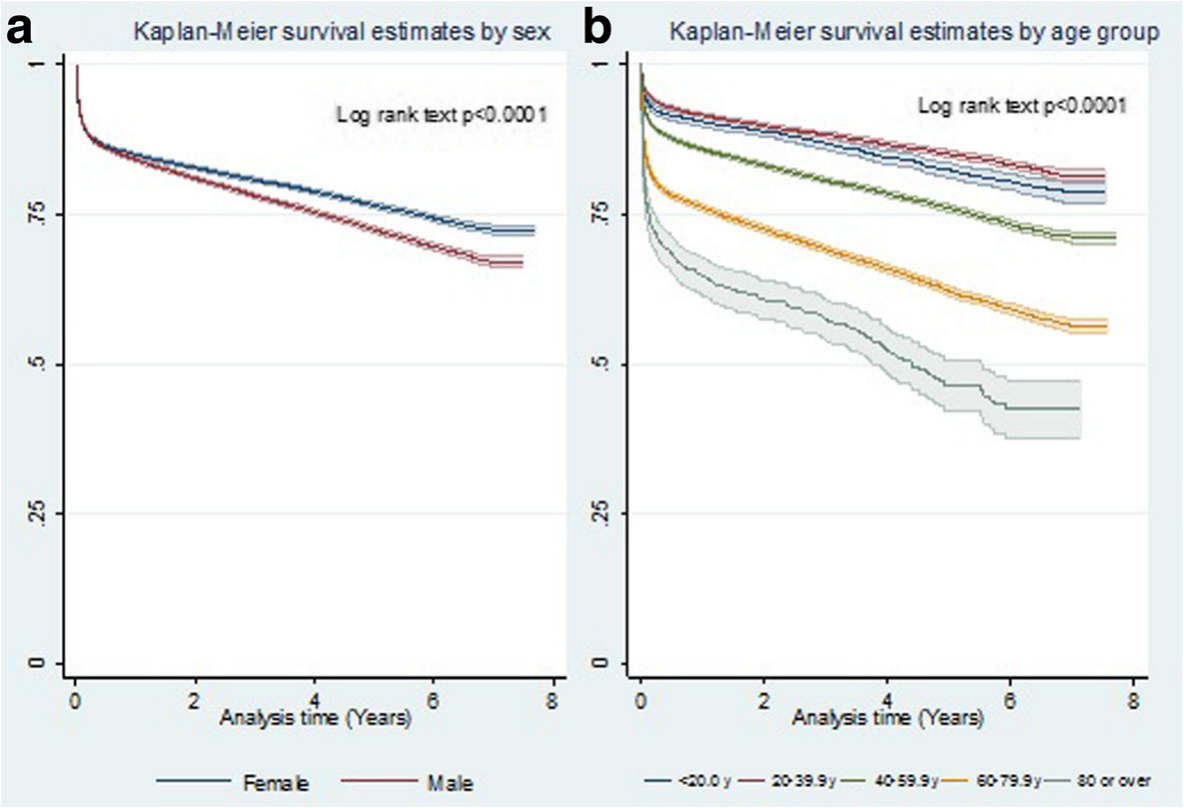
Long-term survival of heart valve surgery patients – 2001 to 2007: **a**) by sex; (**b**) by age group

Survival was observed to differ significantly by sex (Fig. 2a). Long-term survival by sex at year 1 and 5 was, respectively, 85.0% and 76.5% for females and 84.2% and 72.5% for males.

Significant differences by age group are illustrated in Fig. 2b. Survival was worst among patients aged ≥80 years and best in the 20 to 39 year age group. Survival rates after year 1 and 5 of follow-up were, respectively, 64.7%, and 46.4% in the ≥80 year age group and 91.5% and 85.1% in the 20 to 39 year group.

**Fig. 3:**
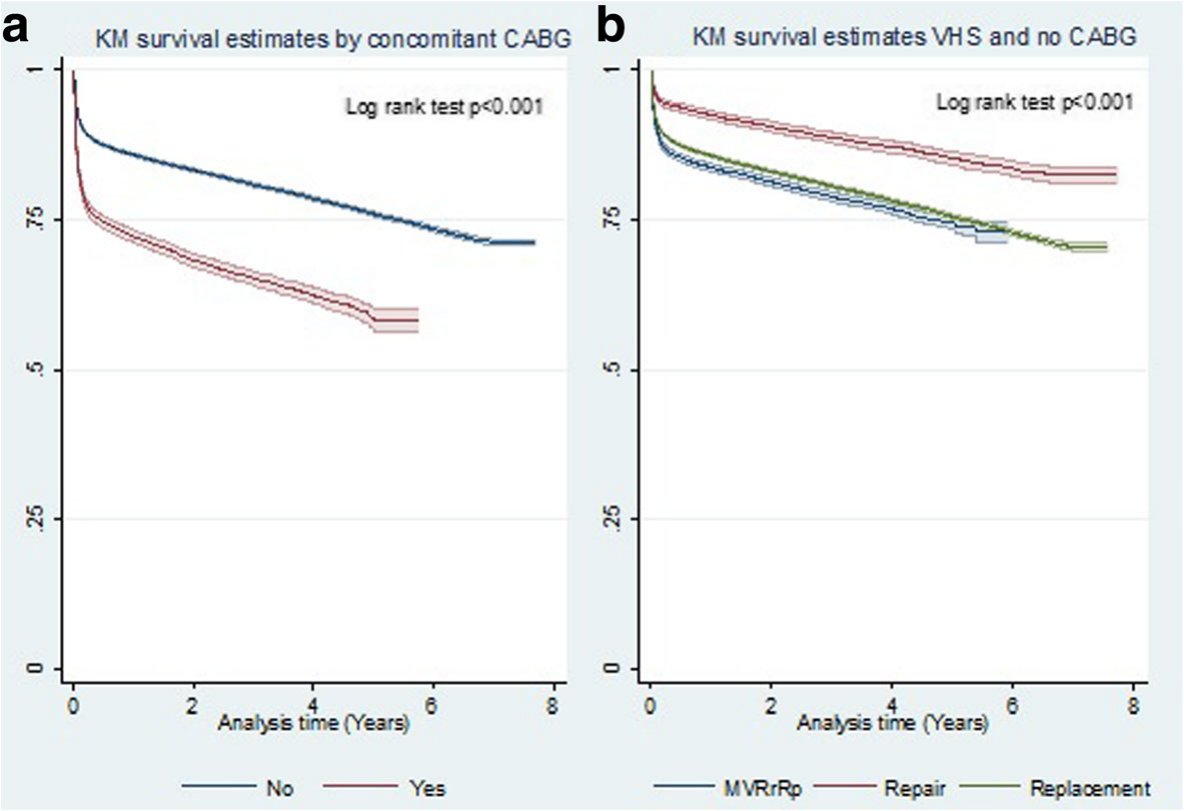
Long-term survival of heart valve surgery patients: **a**) with concomitant coronary artery bypass grafting (CABG); and **b**) heart valve surgery (HVS) with non-concomitant coronary artery bypass grafting (CABG)

Long-term survival rates differed significantly between patients who underwent HVS and concomitant CABG and those who underwent HVS only (Fig. 3a): at years 1 and 5 of follow-up, these were, respectively, 72.1% and 58.1%, against 85.9% and 76.0%.

Figure 3b shows long-term survival of patients submitted to HVS with non-concomitant CABG. Long-term survival rates for VRp and multiple valve surgery were similar at year 1 (85.7% versus 83.8%) and year 5 (75.5% versus 74.4%) of follow-up, while VRr returned highest long-term survival rates (92.5% and 85.3%), respectively.

The proportional hazard assumption was violated for sex and geographical region of residence; accordingly, these variables were stratified in multivariate survival analysis. For the other variables, the proportional hazard assumption was satisfied 30 days after hospital discharge; thus this is the timeframe considered in multivariate analysis. Patients who underwent valve surgery with concomitant CABG showed greater risk than those who underwent valve surgery alone (HR 1.3123; 95%CI 1.2271–1.4033; *p* < 0.0001), while patient age contributed, as secondary factor, to increases in this set (HR 1.0216; 95%CI 1.0203–1.0229; *p* < 0.0001). Non-concomitant CABG patients submitted to valve repair showed lower risk than those with VRp or multiple surgeries (HR 0.6937; 95%CI 0.6296–0.7645; *p* < 0.0001), but age contributed, as a secondary factor, to increases in this set (HR 1.0209; 95%CI 1.0195–1.0222; *p* < 0.0001).

## Discussion

To the best of our knowledge, this retrospective HVS cohort, comprising 78,808 patients, is one of the largest ever published. Sex distribution was balanced. Valve replacement accounted for 69.1% of procedures performed. The most common valve injury was mitral stenosis (38.9% of the total). In 94.7% of mitral stenosis patients, aetiology was rheumatic heart disease. In-hospital mortality was 7.6% and was higher for females who had undergone the concomitant CABG procedure and for the elderly. Overall survival was 69.9% at the end of follow-up time. Survival was worst among elderly, male and concomitant coronary artery bypass grafting patients. Median age was 50.0 years, a lot younger than in studies in developed countries, such as in the Euro Heart Survey on Valvular Heart Disease, where mean age was 64 years [21]. Proportions of valve lesion types and aetiologies were also different from most published studies based on North American and European populations [22–24]. In the “valve lesion identified” subset of the study population, mitral stenosis was the most frequent lesion. RHD was the most prevalent aetiology, accounting for more than half of the subjects studied. This finding is similar to those of other studies in lower- and middle-income countries [10, 25–27].

Rheumatic fever is the leading cause of mitral stenosis worldwide and, in 40% of cases, it presents as a single lesion [28]. In our cohort, the aetiology underlying 94.7% of mitral stenosis lesions was RHD. VRp accounted for more than two thirds of procedures, while multiple valve surgery accounted for 15.48%. Multiple valve surgery was observed more frequently than reported in the literature (3–14%) [29], which probably relates to the prevalence of RHD in our cohort (53.7%).

In developing countries, although repair procedures offer the advantage of averting long-term anticoagulation therapy and achieve better outcomes, VRp is usually performed more than VRr in cases of rheumatic disease [25, 30, 31]. Patients are less likely to be suitable for VRr in developing countries, where waiting times are long and RHD is common; as a result, access to surgery tends to occur at an advanced stage of disease. The likelihood of successful mitral repair is associated with the timing of surgical referral, annual volume and expertise of cardiac surgery centres, underlying aetiologies and type of lesion [26, 27]. In RHD, valve repair may be rendered impossible, even for experienced surgeons, by various degrees of fibrotic process, involvement of *chordae tendineae* and calcification of the mitral leaflets or annulus [25]. In developed countries, at institutions with good performance, valve repair is standard therapy for most patients with mitral regurgitation [32]. Note, however, that the most common aetiologies for valve dysfunction in low- and middle-income countries are different from those studied and often published in developed countries. Accordingly, valve repair entails different levels of challenge and outcomes in these different real settings. Although overall survival has been better among RHD patients than among those with valve lesions with other aetiologies (probably as a result of younger age and fewer comorbidities), valve lesions from rheumatic fever are still a undesirable outcome since RHD is largely avoidable by means of primary and secondary prevention.

Valve injury aetiology was not clearly stated in some groups of our cohort and hence we classified it as “insufficient aetiology description”. The lack of a specific ICD 10.0 code may be one of the underlying causes of insufficient coding. In Brazilian public health databases, the “diagnosis” field draws on the ICD 10.0 codex to classify diseases and other health problems. In the literature, degenerative aetiology plays a very important role in other HVS cohorts [33, 34]. Although this study population is younger, it must be assumed that many patients grouped as “insufficient aetiology description” presented injuries caused by a degenerative process of the heart valves. The median age of these miscoded patients was 60.7 years, in contrast to the remaining group where the median was 44.2 years. Corroborating this assumption, almost 80% of patients over 80 years old were classified as “aetiology insufficiently described”. In addition, the proportion of Brazil’s population over 65 years old was 4.0% in 1980, increasing to 7.4% in 2010. Life expectancy in Brazil increased 10 years in that period and, therefore, aging-related diseases can be expected to emerge [35].

Note also that, in one of the few studies evaluating the burden of HVD in developing countries, the most frequent aetiology was RHD (60.3%), in line with this study, while degenerative valve disease, detected in 15% of patients, was the second most common aetiology [10].

Overall in-hospital mortality was 7.6%, but higher for female patients who had undergone concomitant CABG and for the elderly. Concomitant CABG increases mortality substantially [22, 29, 33]. Here, the study population was younger (age has been identified as an important independent predictor of HVS outcomes in adults) [29, 33, 34]. This study involved more patients than those of Leavitt (1057) and Nicolini (1167), although the number of concomitant CABG surgeries was smaller and involved a younger population. Leavitt and Nicolini reported overall in-hospital mortality of 15.5% and 6.9%, respectively. Hannan (14,190) reported 3.3% for CCV without concomitant CABG and 18.7% for multiple valve surgery with concomitant CABG. Both Hannan and Nicolini observed that in-hospital mortality rates were better for VRr than for replacements, similar what is reported in other studies [22, 36–39]. Ribeiro [40] reported cardiac surgery in-hospital mortality in Brazil as 8.9%, from 2000 to 2003; this was not solely for HVS, however, but included other procedures.

Unlike other published studies [22, 29, 33], this study found better overall long-term survival for women. Although VRr was performed more often in women, it cannot explain this favourable result, because VRr contributed only 6.3% of total procedures. In this study, overall long-term survival rates at 1 and 5 years were 84.6% and 74.5%, which are lower than those reported for a double valve surgery cohort, showing that our outcomes seem to be less satisfactory [29]. Seventy per cent of patients were alive at close of follow-up, which agrees with a previous study with similar procedure mix [41]. Long-term survival for VRr was better than for replacements, as reported in another study [39], but different from Leavitt and Nicolini. The latter author does not supported the theory that, for patients undergoing HVS, VRr is better than replacement. He reported comparable long-term survival at 5 years for VRr and replacement. Different outcomes in different studies can probably be ascribed to cohorts with different demographic characteristics, comorbidities, valve lesion aetiologies, surgeons’ skills and repair techniques, especially in observational studies such as this.

The group of patients who underwent multiple valve surgery comprised a great diversity of clinical features and possible combinations of surgeries, making it difficult to analyse this group in comparison with the VRp group, although they displayed similar results and no statistically significant difference in long-term survival.

Concomitant CABG reduced long-term survival considerably. For patients who underwent concomitant CABG, long-term survival at years 1 and 5 of follow-up was, respectively, 72.1% and 58.1% and, for those who underwent HVS alone, 85.9% and 76.0%.

These rates were similar to those reported in the literature [29, 32, 42, 43]. Hannan and Nicolini have observed that patients who underwent HVS and concomitant CABG were older, had more comorbidities and had more often undergone other previous cardiovascular surgeries. These features could account for lower overall survival.

Taken together with the in-hospital mortality rates and long-term survival found here, length of hospital stay for repair procedures reinforces the assumption that, in Brazil, patients selected for VRr procedures are likely to present better valve and general health status than those submitted to other procedures, which is in line with other studies [34, 39]. Comorbidities and old age predicted lesser likelihood of repair, although good results have been reported for elderly patients undergoing mitral valve surgery, especially mitral VRr, in a large series [34]. Gillinov observed that mitral valve repair was more likely to be performed if valve leaflets were morphologically normal, with functional mitral valve regurgitation, while mitral valve replacement was more often performed in the event of preoperative atrial fibrillation, severe mitral valve stenosis and calcified mitral leaflets or if a mechanical aortic prosthesis was in place [39].

It is clear that old age influences in-hospital mortality, length of hospital stay and long-term survival of patients undergoing HVS. This was also found in this study (OR = 1.0338 for each additional year during hospitalisation). In-hospital mortality was the highest, length of hospital stay, the longest and long-term survival at year 5 of follow-up, the shortest for patients older than 80 years, in agreement with other studies [33, 34, 41, 44].

Length of hospital stay was similar by sex [33] and shorter in patients who died during index procedure admission than in survivors. Most in-hospital deaths (53.9%) occurred up to day 7 of hospitalisation and 25.03% up to day 2. These rates suggest that half of patients died in the operating theatre or in the very early postoperative period. This finding serves to highlight the need to improve the quality of surgical care during the operation or to improve the appropriateness of surgical indications for HVS in Brazil.

The scarcity of long-term survival series drawn from large databases in middle-income countries makes it difficult to compare between studies of populations exposed to similar risk factors and similar patterns of diseases, which reinforces the importance of our findings.

This study was based on a nationwide cohort of patients in the real world setting, which is its main strength. Nevertheless, its conclusions should be read in the light of certain limitations. The two large databases employed offered few clinical variables. This means that we were unable to identify all possible confounding variables. We adjusted for all the available variables, but we should not underestimate the possibility that the omission of important covariates may affect our results.

The limitations of the present study include also the inherent constraints of retrospective, nonrandomised data collection. Although case matching and multivariate analysis can help to account for some differences in the groups, the effects of unmeasured variables affecting selection of therapy may confound the analysis.

## Conclusions

In Brazil at the beginning of the twenty-first century, the most common valve lesion is still mitral stenosis, while the most common underlying aetiology is rheumatic heart disease. Single valve replacement accounts for the overwhelming majority of the procedures performed, whereas only a very small number of patients with heart valve diseases benefited from valve repairs. In-hospital mortality and overall survival rates of patients who have undergone heart valve surgery were less satisfactory than those reported in high-income countries. The findings of this study can help guide decision-making processes in middle-income countries similar to Brazil and those concerned with improving the quality of care.

## Abbreviations

CABG: Coronary artery bypass grafting
CI: Confidence interval
HR: Hazard ratio
HVD: Heart valve disease
HVS: Heart valve surgery
ICD: International Classification of Diseases
IQR: Interquartile range
OR: Odds ratio
RHD: Rheumatic Heart Disease
SIH: Brazilian Hospital Database
SIM: Brazilian National Mortality Database
SUS: Brazilian National Health System
VRp: Valve replacement
VRr: Valve repair
